# Deaths in Philadelphia during the Months of January, February and March, (up to March 23d)

**Published:** 1850-04

**Authors:** James Aitken Meigs

**Affiliations:** Student of Medicine


*Deaths in Philadelphia during the months of January, February and March, (up to March 23d.)* Reported by Mr. JAMES AITKEN MEIGS, Student of Medicine.

(To be continued monthly.)

| Diseases. | JANUARY. Adults. | JANUARY. Chil. | FEBRUARY. Adults. | FEBRUARY. Chil. | MARCH. Adults. | MARCH. Chil. | Total. Ad. | Total. Ch. |
|---|---|---|---|---|---|---|---|---|
| Abscess, | 0 | 1 | 1 | 0 | 0 | 0 | 1 | 1 |
| " of liver, | 1 | 0 | 0 | 0 | 0 | 0 | 1 | 0 |
| " lungs | 1 | 0 | 1 | 1 | 1 | 0 | 3 | 1 |
| " neck, | 0 | 0 | 0 | 1 | 0 | 1 | 0 | 2 |
| " pleura, | 0 | 1 | 0 | 0 | 0 | 0 | 0 | 1 |
| Anæmia, | 0 | 0 | 0 | 1 | 0 | 0 | 0 | 1 |
| Angina Pectoris, | 0 | 0 | 2 | 1 | 0 | 0 | 2 | 1 |
| Apoplexy, | 7 | 0 | 6 | 0 | 7 | 1 | 20 | 1 |
| Aphthæ, | 0 | 0 | 0 | 0 | 0 | 1 | 0 | 1 |
| Asphyxia, | 0 | 0 | 0 | 2 | 0 | 1 | 0 | 3 |
| Asthma, | 0 | 0 | 0 | 0 | 1 | 0 | 1 | 0 |
| Burns and scalds, | 3 | 2 | 3 | 2 | 0 | 2 | 6 | 6 |
| Cachexia, | 1 | 0 | 0 | 0 | 1 | 0 | 2 | 0 |
| Cancer, | 3 | 0 | 0 | 0 | 1 | 0 | 4 | 0 |
| " of breast, | 1 | 0 | 0 | 0 | 1 | 0 | 2 | 0 |
| " liver, | 0 | 0 | 1 | 0 | 0 | 0 | 1 | 0 |
| " pylorus, | 1 | 0 | 0 | 0 | 0 | 0 | 1 | 0 |
| " stomach, | 2 | 0 | 1 | 0 | 0 | 0 | 3 | 0 |
| " uterus, | 4 | 0 | 0 | 0 | 1 | 0 | 5 | 0 |
| Casuality, | 4 | 1 | 1 | 1 | 3 | 1 | 8 | 3 |
| Child-bed, | 1 | 0 | 0 | 0 | 1 | 0 | 2 | 0 |
| Cholera infantum, | 0 | 0 | 0 | 1 | 0 | 2 | 0 | 3 |
| " morbus, | 1 | 0 | 0 | 0 | 0 | 0 | 1 | 0 |
| Compression of brain, | 0 | 1 | 0 | 0 | 0 | 0 | 0 | 1 |
| Congestion of brain, | 3 | 3 | 0 | 2 | 5 | 2 | 8 | 7 |
| " heart, | 0 | 0 | 0 | 1 | 0 | 0 | 0 | 1 |
| " lungs, | 1 | 6 | 2 | 1 | 1 | 4 | 4 | 11 |
| Convulsions, | 2 | 28 | 0 | 26 | 0 | 29 | 2 | 83 |
| " puerperal, | 0 | 0 | 0 | 1 | 0 | 0 | 0 | 1 |
| Croup, | 0 | 17 | 0 | 17 | 0 | 7 | 0 | 41 |
| Cyanosis, | 0 | 3 | 0 | 1 | 0 | 0 | 0 | 4 |
| Debility, | 3 | 5 | 4 | 5 | 5 | 8 | 12 | 18 |
| Diarrhœa, | 1 | 3 | 2 | 3 | 5 | 3 | 8 | 9 |
| Disease of brain, | 2 | 11 | 2 | 4 | 1 | 4 | 5 | 19 |
| " bowels and stomach, | 0 | 1 | 0 | 0 | 0 | 1 | 0 | 2 |
| " heart, | 9 | 3 | 4 | 0 | 5 | 7 | 18 | 10 |
| " kidneys, | 0 | 0 | 1 | 0 | 0 | 0 | 1 | 0 |
| " liver, | 1 | 0 | 2 | 1 | 1 | 1 | 4 | 2 |
| " lungs, | 1 | 1 | 1 | 2 | 0 | 1 | 2 | 4 |
| " prostate gland, | 1 | 0 | 0 | 0 | 0 | 0 | 1 | 0 |
| " spine, | 0 | 0 | 0 | 0 | 0 | 1 | 0 | 1 |
| Dropsy, | 14 | 2 | 1 | 3 | 7 | 1 | 22 | 6 |
| " of abdomen, | 1 | 1 | 2 | 0 | 2 | 1 | 5 | 2 |
| " breast, | 4 | 4 | 3 | 1 | 1 | 3 | 8 | 8 |
| " head, | 1 | 15 | 0 | 14 | 1 | 27 | 2 | 56 |
| " heart, | 0 | 0 | 0 | 0 | 0 | 2 | 0 | 2 |
| Drowning, | 3 | 0 | 1 | 0 | 3 | 0 | 7 | 0 |

| Diseases. | January. Adults. | January. Chil. | February. Adults. | February. Chil. | March. Adults. | March. Chil. | Total. Ad. | Total. Ch. |
|---|---|---|---|---|---|---|---|---|
| Dysentery, | 3 | 6 | 5 | 1 | 3 | 4 | 11 | 11 |
| Dysmenorrhœa, | 0 | 1 | 0 | 0 | 0 | 0 | 0 | 1 |
| Dyspepsia, | 1 | 0 | 0 | 0 | 0 | 0 | 1 | 0 |
| Effusion on brain, | 0 | 5 | 0 | 3 | 0 | 3 | 0 | 11 |
| " lungs, | 0 | 0 | 1 | 1 | 0 | 0 | 1 | 1 |
| Enlargement of heart, | 0 | 0 | 3 | 1 | 1 | 0 | 4 | 1 |
| " liver, | 0 | 1 | 0 | 1 | 0 | 0 | 0 | 2 |
| " ovary, | 1 | 0 | 0 | 0 | 0 | 0 | 1 | 0 |
| Epilepsy, | 1 | 1 | 1 | 0 | 0 | 2 | 2 | 3 |
| Erysipelas, | 1 | 3 | 0 | 4 | 3 | 1 | 4 | 8 |
| Exposure, | 0 | 1 | 0 | 0 | 0 | 0 | 0 | 1 |
| Fever, | 1 | 2 | 0 | 0 | 2 | 1 | 3 | 3 |
| " bilious, | 1 | 1 | 0 | 1 | 0 | 3 | 1 | 5 |
| " catarrhal, | 0 | 1 | 0 | 0 | 0 | 0 | 0 | 1 |
| " congestive, | 0 | 0 | 0 | 1 | 1 | 0 | 1 | 1 |
| " continued, | 0 | 0 | 0 | 0 | 1 | 0 | 1 | 0 |
| " hectic, | 0 | 0 | 0 | 2 | 0 | 0 | 0 | 2 |
| " intermittent, | 0 | 1 | 0 | 0 | 0 | 0 | 0 | 1 |
| " nervous, | 0 | 0 | 0 | 0 | 1 | 0 | 1 | 0 |
| " puerperal, | 0 | 0 | 3 | 1 | 4 | 0 | 7 | 1 |
| " remittent, | 3 | 1 | 0 | 0 | 0 | 1 | 3 | 2 |
| " scarlet, | 2 | 54 | 1 | 34 | 1 | 49 | 4 | 137 |
| " typhoid, | 6 | 1 | 5 | 0 | 4 | 3 | 15 | 4 |
| " typhus, | 4 | 2 | 5 | 0 | 3 | 3 | 12 | 5 |
| Fracture of skull, | 1 | 0 | 0 | 0 | 0 | 0 | 1 | 0 |
| Gangrene, | 0 | 0 | 1 | 0 | 0 | 0 | 1 | 0 |
| Hemorrhage, | 0 | 1 | 0 | 0 | 0 | 1 | 0 | 2 |
| " from aorta, | 1 | 0 | 0 | 0 | 0 | 0 | 1 | 0 |
| " " bowels, | 0 | 1 | 0 | 1 | 1 | 0 | 1 | 2 |
| " " lungs, | 2 | 1 | 0 | 0 | 3 | 1 | 5 | 2 |
| " " uterus, | 0 | 0 | 1 | 0 | 0 | 0 | 1 | 0 |
| Icterus, | 0 | 0 | 0 | 1 | 0 | 1 | 0 | 2 |
| Ileus, | 0 | 0 | 1 | 0 | 0 | 0 | 1 | 0 |
| Inanition, | 1 | 2 | 0 | 1 | 0 | 2 | 1 | 5 |
| Inflam. of brain, | 2 | 15 | 3 | 7 | 3 | 16 | 8 | 38 |
| " breast, | 0 | 2 | 1 | 0 | 1 | 2 | 2 | 4 |
| " bronchi, | 4 | 26 | 5 | 14 | 3 | 12 | 12 | 52 |
| " fauces, | 0 | 1 | 0 | 0 | 0 | 1 | 0 | 2 |
| " heart, | 0 | 0 | 2 | 0 | 1 | 2 | 3 | 2 |
| " kidneys, | 1 | 0 | 0 | 0 | 0 | 0 | 1 | 0 |
| " larynx, | 1 | 1 | 0 | 0 | 0 | 1 | 1 | 2 |
| " liver, | 4 | 0 | 1 | 0 | 3 | 0 | 8 | 0 |
| " lungs, | 12 | 25 | 8 | 24 | 21 | 21 | 41 | 70 |
| " peritoneum, | 6 | 2 | 1 | 3 | 5 | 2 | 12 | 7 |
| " pleura, | 1 | 0 | 2 | 0 | 0 | 0 | 3 | 0 |
| " salivary glands | 1 | 0 | 0 | 0 | 0 | 0 | 1 | 0 |
| " stomach and bowels, | 7 | 5 | 7 | 3 | 7 | 7 | 21 | 15 |
| " trachea, | 0 | 0 | 0 | 0 | 0 | 1 | 0 | 1 |
| " uterus, | 0 | 0 | 0 | 0 | 1 | 0 | 1 | 0 |
| Influenza, | 0 | 1 | 0 | 0 | 0 | 0 | 0 | 1 |
| Intemperance and exposure, | 3 | 0 | 3 | 0 | 5 | 0 | 11 | 0 |
| Ischuria, | 0 | 0 | 0 | 0 | 0 | 1 | 0 | 1 |
| Lupus Exedens, | 0 | 0 | 0 | 0 | 1 | 0 | 1 | 0 |
| Malformation, | 0 | 1 | 0 | 2 | 0 | 0 | 0 | 3 |

| Diseases. | January. Adults. | January. Chil. | February. Adults. | February. Chil. | March. Adults. | March. Chil. | Total. Ad. | Total. Ch. |
|---|---|---|---|---|---|---|---|---|
| Malformation of heart, | 0 | 0 | 0 | 1 | 0 | 1 | 0 | 2 |
| " throat, | 0 | 0 | 0 | 1 | 0 | 0 | 0 | 1 |
| Mania-a-potu, | 4 | 0 | 2 | 0 | 3 | 1 | 9 | 1 |
| Marasmus, | 2 | 3 | 0 | 6 | 3 | 5 | 5 | 14 |
| Measles, | 0 | 3 | 0 | 3 | 0 | 12 | 0 | 18 |
| Mortification, | 1 | 0 | 0 | 0 | 0 | 0 | 1 | 0 |
| Mortification of leg, | 0 | 0 | 0 | 0 | 1 | 0 | 1 | 0 |
| " lungs, | 1 | 0 | 0 | 0 | 0 | 0 | 1 | 0 |
| Neuralgia, | 1 | 0 | 0 | 0 | 0 | 1 | 1 | 1 |
| Obstruction of bowels, | 0 | 0 | 0 | 0 | 0 | 1 | 0 | 1 |
| Œdema of lungs, | 0 | 0 | 0 | 0 | 0 | 1 | 0 | 1 |
| Old age, | 11 | 0 | 10 | 0 | 16 | 0 | 37 | 0 |
| Ossification of heart, | 0 | 1 | 0 | 0 | 0 | 0 | 0 | 1 |
| " valves of heart, | 0 | 0 | 1 | 0 | 0 | 0 | 1 | 0 |
| Palsy, | 8 | 0 | 3 | 1 | 4 | 0 | 15 | 1 |
| " of brain, | 0 | 0 | 0 | 1 | 0 | 0 | 0 | 1 |
| " lungs, | 0 | 0 | 0 | 0 | 1 | 0 | 1 | 0 |
| Parotitis, | 1 | 0 | 0 | 1 | 0 | 0 | 1 | 1 |
| Pertussis, | 0 | 2 | 0 | 2 | 0 | 8 | 0 | 12 |
| Phthisis pulmonalis, | 63 | 12 | 49 | 12 | 57 | 13 | 169 | 37 |
| Phlebitis, | 1 | 0 | 0 | 0 | 1 | 0 | 2 | 0 |
| Pneumothorax, | 0 | 0 | 0 | 0 | 1 | 0 | 1 | 0 |
| Poisoning, | 0 | 0 | 1 | 0 | 1 | 0 | 2 | 0 |
| Prolapsus ani, | 0 | 1 | 0 | 0 | 1 | 1 | 1 | 2 |
| Purpura, | 0 | 1 | 0 | 0 | 0 | 0 | 0 | 1 |
| Rheumatism, | 2 | 0 | 1 | 0 | 0 | 0 | 3 | 0 |
| Scrofula, | 1 | 3 | 0 | 1 | 0 | 7 | 1 | 11 |
| Small pox, | 0 | 2 | 1 | 2 | 0 | 1 | 1 | 5 |
| Softening of brain, | 0 | 1 | 0 | 0 | 1 | 0 | 1 | 1 |
| " stomach, | 0 | 0 | 0 | 0 | 0 | 1 | 0 | 1 |
| Still born, | 0 | 34 | 0 | 27 | 0 | 39 | 0 | 100 |
| Strangulation, | 0 | 1 | 0 | 0 | 0 | 0 | 0 | 1 |
| Suicide, | 1 | 0 | 1 | 0 | 1 | 0 | 3 | 0 |
| Syncope, | 0 | 0 | 1 | 0 | 0 | 0 | 1 | 0 |
| Tabes mesenterica, | 0 | 1 | 0 | 0 | 0 | 0 | 0 | 1 |
| Teething, | 0 | 1 | 0 | 2 | 0 | 0 | 0 | 3 |
| Tuberculosis, | 2 | 0 | 0 | 0 | 1 | 1 | 3 | 1 |
| Tumor, | 0 | 0 | 1 | 0 | 0 | 0 | 1 | 0 |
| " of brain, | 0 | 0 | 0 | 0 | 1 | 0 | 1 | 0 |
| Ulceration of bowels, | 2 | 0 | 0 | 0 | 0 | 0 | 2 | 0 |
| Ulcered sore throat, | 0 | 0 | 0 | 0 | 0 | 2 | 0 | 2 |
| Unknown, | 2 | 9 | 1 | 4 | 0 | 11 | 3 | 24 |
| Violence, | 0 | 1 | 0 | 0 | 0 | 0 | 0 | 1 |
| Wounds, | 2 | 0 | 0 | 0 | 0 | 0 | 2 | 0 |
| | | | | | | | 651 | 971 |
| Total, | | | | | | | 1,622 | |

| | Adults. | Children. |
|---|---|---|
| January, | 253 | 350 |
| February, | 173 | 262 |
| March, | 225 | 359 |
| | 651 | 971 |

Of the foregoing the ages were as follows:—

| | | | | |
|---|---|---|---|---|
| Under | 1 year, | | | 451 |
| From | 1 | to | 2, | 162 |
| | 2 | - | 5, | 195 |
| | 5 | - | 10, | 97 |
| | 10 | - | 15, | 22 |
| | 15 | - | 20, | 43 |
| | 20 | - | 30, | 155 |
| | 30 | - | 40, | 134 |
| | 40 | - | 50, | 111 |
| | 50 | - | 60, | 83 |
| | 60 | - | 70, | 59 |
| | 70 | - | 80, | 66 |
| | 80 | - | 90, | 39 |
| | 90 | - | 100, | 4 |
| | 100 | - | 110, | 1 |
| | | | | 1622 |

Included in this number, are 94 from the Almshouse, 132 people of color, and 15 from the surrounding country.

